# Nominalist dispositional essentialism

**DOI:** 10.1007/s11229-022-03588-z

**Published:** 2022-04-15

**Authors:** Lisa Vogt

**Affiliations:** 1 LOGOS, University of Barcelona, Barcelona, Spain; 2 Department of Philosophy, Freie Universität Berlin, Berlin, Germany; 3 Faculty of Theology, University of Lucerne, Lucerne, Switzerland

**Keywords:** Dispositional Essentialism, Nominalism, Essence, Generic essence, Powers, Laws of nature, Causal Nominalism

## Abstract

Dispositional Essentialism, as commonly conceived, consists in the claims that at least some of the fundamental properties essentially confer certain causal-nomological roles on their bearers, and that these properties give rise to the natural modalities. As such, the view is generally taken to be committed to a realist conception of properties as either universals or tropes, and to be thus incompatible with nominalism as understood in the strict sense. Pace this common assumption of the ontological import of Dispositional Essentialism, the aim of this paper is to explore a nominalist version of the view, Austere Nominalist Dispositional Essentialism. The core features of the proposed account are that it eschews all kinds of properties (be they universals, tropes, or sets of particulars), takes certain predicative truths as fundamental, and employs the so-called generic notion of essence. As I will argue, the account is significantly closer to the core idea behind Dispositional Essentialism than the only nominalist account in the vicinity of Dispositional Essentialism that has been offered so far—Ann Whittle’s (2009) Causal Nominalism—and is immune to crucial problems that affect this view.

## Introduction

According to *Dispositional Essentialism* (‘DE’), as commonly conceived, natural modality is intimately tied to the essences of properties. On this view, at least some of the fundamental properties are powers, that is properties which essentially confer certain causal-nomological roles on the objects that instantiate them. Thus, for instance, a dispositional essentialist might maintain that it is part of the essence of the property of unit negative electric charge that objects with this property repel other negatively charged particles with a certain magnitude. The dispositional essentialist then conceives of these dispositional essences as the metaphysical sources of the different kinds of natural modality, such as causation, counterfactual connections and the laws of nature.^1^

DE seems to go hand in hand with a realist conception of properties as irreducible property universals or tropes. Moreover, there are reasons to think that the ontological commitment of DE goes even deeper: Virtually all the accounts of DE in the literature are based on a universals-account of properties, and it has been argued that DE is incompatible with both trope views (Tugby 2013) and Aristotelian views of universals (Dumsday 2013; Tugby 2013, 2015; Yates 2016). If these arguments were sound, this would leave the Platonist view of universals as the sole option—a view that many people in the debate on natural modality find hard to swallow.

In her ‘Causal Nominalism’ (2009), however, Ann Whittle showed that a nominalist account in the vicinity of DE can be given. Her account eschews any commitment to irreducible property universals and tropes, and yet shares DE’s basic idea that properties and natural modality are intimately tied to one another. But while Causal Nominalism constitutes an important position in conceptual space, the view has not found further advocates in the debate, arguably because it departs quite substantially from the core tenets of DE and is affected by serious systematic problems (cf. Tugby 2016). Given that Causal Nominalism is the only account of nominalist DE (i.e., the combination of DE and nominalism) that has been offered thus far, it might seem that going nominalist is not a promising option for the dispositional essentialist.

The aim of this paper is to argue to the contrary: Going nominalist should be considered as a live option for the dispositional essentialist that is worthy of further consideration. I will explore a novel version of nominalist DE, *Austere Nominalist Dispositional Essentialism*. As I will argue, the view allows us to mimic the core tenets of the standard reified (i.e., property realist) versions of DE within a nominalist set-up extremely closely, and it is immune to the objections raised by Matthew Tugby against Causal Nominalism.

I start out by introducing the relevant background framework regarding essence, grounding, and fundamentality (§2). I then outline the core tenets of the standard reified accounts of DE (§3). In the central part of the paper, I develop and further explore the account of Austere Nominalist Dispositional Essentialism, my proposal for nominalist DE (§4). Finally, I end with some concluding remarks concerning the dialectical upshot of the results (§5).

## Background framework

This section introduces the background framework that I will use in what follows. Since some of these claims rely on resources that are only available in formal languages and can be merely approximated in natural language, I will also always provide formal regimentations of the notions under consideration.

### Essence

The commonly employed notion of essence in contemporary metaphysics is that of *objectual* essence. On this understanding, essence concerns features that pertain to the very nature of a certain entity, or, in other words, features that tell us what this entity is at its very core. Thus, for instance, we may maintain that it is essential to Socrates that he be human, that it is essential to the singleton {Socrates} that it have Socrates as a member, or that it is essential to God that she be wise.

It was commonplace in analytic metaphysics for a long time to analyze essence in modal terms. On such an understanding, for Socrates to be essentially human would simply reduce to him being necessarily human, or, alternatively, human in all worlds in which he exists. As Michael Dunn (1990) and Kit Fine (1994a) showed, however, this account of essence is defective in the sense that it fails to capture what philosophers commonly have in mind when they say that an entity is essentially thus-and-so. To use the commonly cited Finean example, while Socrates is necessarily a member of his singleton set, this is not an essential feature of him.^2^ In reaction to cases like that of singleton {Socrates}, Fine proposes that we reverse the order of explanation: We take essence as primitive, and analyze metaphysical modality in terms of it. The common means of formalizing objectual essentialist claims is the $\square _{\cdot }\cdot$-operator. It takes a nominal term for an entity—such as ‘*e*’—and a sentence—such as ‘*p*’—as its input, and yields anther sentence—‘$\Box _{e} p$’—as its output. For instance, with this notation, the previous essentialist claim about Socrates could then be expressed in the following way:

      $\Box _{Socrates}$(Socrates is human).

A crucial distinction that will become relevant in what follows is that between *immediate* essence on the one hand, and *mediate* essence on the other. While the immediate essence of some object only ‘include[s] that which has a direct bearing on the nature of the object’ (Fine 1994b, p. 61), the mediate essence of an object also includes features that, so to speak, arise due to the chaining of immediate essence. For instance, to borrow again an example from Fine, while it is immediately essential to Socrates’ singleton that it have Socrates as a member, and immediately essential to Socrates that he be human, it is only mediately essential to the singleton that it have a member that is human.

### Grounding

Grounding is commonly conceived as a form of non-causal determination which gives rise to a layered structure of reality and underlies metaphysical explanations.^3^ Some paradigmatic examples of candidate grounding claims are: (a) Mental truths obtain in virtue of physical truths; (b) The fact that snow is white and the fact that grass is green jointly ground the fact that snow is white and grass is green; (c) Singleton {Socrates} exists in virtue of the existence of Socrates; (d) The ocean is blue because it is azure. It is generally assumed that conjunctions are grounded in their conjuncts, disjunctions in their disjuncts, and universal as well as particular generalizations in their instances, plus maybe totality truths. Moreover, grounding is commonly taken to be transitive and asymmetric (and hence also irreflexive).^4^

In what follows, I will adopt an *operationalist* formalization of grounding claims, i.e., regiment grounding claims in terms of a sentential operator, as we have it in the example claim (d).^5^ In formal language, I will employ the operator ‘<’, which goes in the opposite direction of ‘because’. Thus, for instance we have:

      The ocean is azure < The ocean is blue.

      Snow is white, grass in green < Snow is white and grass is green.

To enhance readability, I will nevertheless help myself to formulations such as ‘that the ball is maroon grounds that it is red’, ‘physical truths ground mental truths’ etc., in non-regimented natural language, which are shorthand for sentences of the form ‘$p < q$’ in regimented language.

### Fundamentality

As is commonplace for proponents of grounding, I shall conceive of a truth’s being fundamental as its being ungrounded. Corresponding to the operationalist formulation of grounding, I will thus also employ a sentential operator for truth-fundamentality, i.e., ‘it is fundamentally the case that _’ or ‘$\mathbb {F}$’ in formal language:

      $$\mathbb {F}p$$ iff$$_{\tiny {\text{ def }}}$$ $$ p \; \& \; \lnot \exists q_{1}, q_{2},... (q_{1}, q_{2},... < p)$$.

Besides this notion of truth-fundamentality, there is also a second notion of fundamentality that is relevant in the context of DE, viz., that of *entity* fundamentality, which figures in DE’s claims regarding the essences of *fundamental properties*. In contrast to the notion of truth-fundamentality, however, there is no general consensus about how we should understand this notion. In this paper, I will restrict attention without further argument to two options that I take to be particularly promising in the case of DE.^6^ That being said, I do think that all the different extant proposals for accounts of property fundamentality are in principle amenable to nominalist reconstruction, and that restriction to these two options is thus merely for purposes of presentation. The first promising way to understand the notion of property fundamentality is to regard the notion as a primitive, along the lines of e.g. David Lewis’ (1983) and Ted Sider’s (2011) accounts of perfect naturalness and perfect structuralness, respectively, or Jessica Wilson’s (2014) account. The second option would be to define entity fundamentality out of truth fundamentality. Going this route, we may regard a property as fundamental iff it is, so to speak, fundamentally instantiated—i.e., iff at least one truth that concerns the instantiation of this property is fundamental in the sense outlined before. Let us use ‘$\mathscr {F}$’ as a predicate for property-fundamentality, and ‘*I*’ for ‘instantiates’ (or ‘exemplifies’). Then this option would amount to:

      $\mathscr {F} (F$ness) iff$_{\tiny {\text{ def }}}$ $\exists x \; \mathbb {F} (xIF$ness).^7^

With the general framework from this section at our disposal, let us now turn to the discussion of dispositional essentialism.

## Standard reified dispositional essentialism

In this section, I will present the three general claims which, I take it, form the common core of most accounts of DE that have been proposed in the literature thus far. I shall refer to the combination of these claims as ‘Standard (reified) Dispositional Essentialism’, or ‘SDE’ for short. While the claims that make up SDE are not beyond controversy, they are endorsed by the majority of dispositionalist essentialists, and can be regarded as jointly forming the bare bones for a paradigmatic account of DE. In addition to presenting SDE’s general claims in abstraction, I shall also illustrate their application on a concrete toy example, in order to provide claims which will later allow me to illustrate the ‘translation’ of SDE into the nominalist account in a precise way. Once it is clear how the nominalist can recast the example claims discussed here, it will also be clear how they can then recast other dispositional essentialist claims.

*The Ontological Claim*

The first of the claims that jointly make up SDE is that there are irreducible property universals or tropes, or both of them.^8^ This claim is, of course, not specific to DE, but rather, common to all realist accounts of properties.

*The Essentialist Claim*

The first distinctively dispositionalistic claim of SDE is that at least some of the fundamental properties are so-called powers, that is, properties with dispositional essences.^9^ At the most general level, a dispositional essence might be characterized as an essence that ‘specifies’ the nomic role that the property confers on its bearers: viz. which kind of causal, counterfactual, or dispositional modalities hold of objects that instantiate the corresponding property. Let us use the symbol ‘$\mathscr {D}$’ as a placeholder for the predicate ‘is a property with a dispositional essence’. Then, the Essentialist Claim can be expressed as follows:

      $$ \exists x (\mathscr {F}x \; \& \; \mathscr {D}x)$$.^10^

I will use the case of the property of unit negative electric charge as my toy-example throughout the paper. To simplify formulations, I shall simply use the term ‘charge’ to denote this property, and speak of an object’s ‘being charged’ when that object is unit negatively charged. On the counterfactual conception, an essentialist claim regarding this property might then for instance be taken to be:

It is essential to charge that:

If some object *x* is charged, then, for all objects *y* and magnitudes *u*, *v*:

If *x* were at distance *u* from *y* and *y* had charge *v*, *x* would exert a force of $\epsilon \frac{e \cdot v}{u^{2}}$.

Let us use ‘*x* plays role *R*’/‘*Rx*’ as an abbreviation for the claim that the above embedded universally quantified counterfactual modality holds of *x*, ‘*F*’ as a placeholder for ‘is charged’ and ‘*F*ness’ as a placeholder for ‘charge’. Then, the aforementioned essentialist claim can be formalized as follows:

$$\Box _{Fness} \forall x (Fx \rightarrow Rx)$$.^11^,^12^

*The Explanatory Claim*

The second characteristic claim of DE is that we can provide metaphysical explanations for natural modality in terms of powers.

One natural interpretation would be to regiment this claim in terms of grounding: i.e., as maintaining that natural modalities are grounded in certain truths regarding the essences and/or instantiations of powers. Now, while I think that there are in principle also other ways in which the relevant explanatory connection could be construed, I do take this grounding construal to be the natural default option. I will thus focus on it here, leaving the discussion of potential alternatives for other occasions.^13^

Here is a toy-example of how such explanations might look. In our case of charge, what is to be explained are both the fact that *every* charged object *x* plays role *R*, and the fact that *some specific* electron *a* (which in fact is charged) plays role *R*. Now, in the former general case, a plausible candidate for an explanans is the essentialist truth regarding charge on its own:

It is essential to charge that every charged object plays role *R* < every charged object plays role *R*.

$$\Box _{Fness} (\forall x (Fx \rightarrow Rx)) < \forall x (Fx \rightarrow Rx)$$.^14^

In the particular case, by contrast, the essentialist truth will not suffice on its own to make it the case that this particular object *a* plays role *R*—we also need to invoke the fact that *a* is indeed charged to acquire a full explanation:

It is essential to charge that every charged object plays role *R*, *a* is charged < *a* plays role *R*.^15^

$$ \Box _{Fness} (\forall x (Fx \rightarrow Rx)), Fa < Ra$$.

How about the laws of nature? Here, matters are less straightforward, and proponents of SDE have various options at their disposal. One option would simply be to identify the laws with the relevant dispositional essentialist truths. Alternatively, they might identify the laws with certain universal generalizations that (roughly) reflect the relevant essentialist truths (Bird 2007), or with generalizations that provide the best systematization of the fundamental property distributions in either the actual or in all possible worlds (Demarest 2017; Kimpton-Nye 2017; Williams 2019). Or they might even choose to dispense with laws all together (Mumford 1998).

With this sketch of the three claims of the common, reified view of DE in the background, it is now time for us to turn to the core question of the paper: Is it possible to preserve the core tenets of DE within a nominalist setting? And if so, what is the best way of doing so?

## Austere Nominalist Dispositional Essentialism

Any account of nominalist DE has to reject the Ontological Claim of SDE according to which there are irreducible properties—after all, that this claim be rejected is the characteristic tenet of nominalism. But, nevertheless, the account has to preserve the dispositionalistic import of SDE. And, arguably, in order to do so, the account has to either preserve or mimic the two distinctively dispositionalistic claims of SDE, i.e., the Essentialist Claim and the Explanatory Claim. That is, we should expect of any ‘full-blown’ account of nominalist DE that it offers us nominalistically acceptable substitutes for these two claims.

As I will show in the next subsection, however, we encounter a prima facie very general difficulty when trying to find a nominalistic substitute for dispositional essence and thus keeping the Essentialist Claim. This difficulty would also seem to jeopardize the possibility of providing a substitute for the Explanatory Claim. I will begin my discussion of nominalist DE by delineating this difficulty (§4.1). Then, I will go on to propose a way out (§4.2), and subsequently construct my proposal for an account of nominalist DE, Austere Nominalist Dispositional Essentialism, on the basis of this discussion (§4.3). Finally, I will say more about how the proposed account compares to SDE and other views on natural modality in the literature (§4.4), and argue that it is immune to some crucial problems that affect Causal Nominalism, the only other extant proposal for nominalist DE (§4.5).

### Recovering the essentialist claim: a dilemma?

Given that nominalism eschews any commitment to irreducible properties, a nominalist has in principle two options at her disposal. First, she can endorse an austere ontology by eliminating properties entirely from her ontology. Or, second, she can opt for ‘proxy’ properties, that is, reconstruct properties as entities of other categories. But no matter which of the two options the proponent of nominalist DE chooses to adopt, it would seem that she is unable to offer a nominalist substitute for the Essentialist Claim.

The difficulty comes out most clearly in the case in which the nominalist adopts an austere ontology. For then, we have no properties that could serve as bearers of essence. And it does not seem that we can offer a convincing substitute of the claim by relying on the essence of non-properties. First, if we were to instead rely on the essence of representational entities such as concepts, we would not get connections ‘out there in the world’, as we would wish to, but merely connections regarding the way in which we conceive of the world. Nor can we take the essence-bearers to be particular objects, on pain of ending up with an entirely different view. And finally, we also cannot take them to be facts—such as the fact [*a* is charged] (even when leaving potential nominalist scruples about ontological commitment to facts to the side). For facts would provide us with merely particular, rather than the desired general connections. We would have a distinct essence for the fact [$a_{1}$ is charged], the fact [$a_{2}$ is charged], and so on. And if these were the only essentialist truths we had, we would lack any deeper explanation of why there is a striking regularity regarding the essence of all these facts: i.e., the regularity that it is essential to the fact [$a_{1}$ is charged] that, if it were to obtain, so would the fact [$Ra_{1}$]; that it is essential to the fact [$a_{2}$ is charged] that, if it were to obtain, so would the fact [$Ra_{2}$]; etc. A dispositionalistic account that relied on the essence of facts would thus greatly deviate from SDE, and fare substantially worse in terms of explanatory and unifying power.

It would thus seem natural to think that the way to go for the proponent of nominalist DE is to instead adopt the second option of admitting proxy properties. The common way of reconstructing properties is in terms of their (possible) instances, e.g. in the case of charge, all the (possible) charged objects. And given that the dispositional essentialist wants to connect properties with causal-nomological roles, it would seem natural to identify properties with sets of (possible) objects that play the corresponding roles.^16^ For instance, on such an account, the property of charge might plausibly be taken to be the set of all (possible) objects that play role *R*. (And, as we will see in §4.4, this is exactly the account favored by Whittle.) At first glance, this account may look like an option that is congenial to the dispositionalistic picture. On closer examination, however, it does not allow us to preserve the Essentialist Claim: While the account would indeed imply that there are *necessary* connections between properties and causal-nomological roles, on the commonly endorsed conception of sets, it would not be able to guarantee that there are the right *essential* connections between them. To see this, let us first take a closer look at the essences of sets in general, before then turning to the case of proxy-properties DE.

On the commonly assumed conception of sets, sets are simply collections of specific objects. More precisely, on this conception, the only features that pertain to the *immediate essence* of a given set are its members, plus that it be a set. For instance, the immediate essence of the set $\{$Barack Obama, Donald Trump$\}$ is exhausted by the fact that it contain Barack Obama and Donald Trump, and the fact that it be a set.

If we shift focus from this narrow conception of immediate essence to the broader conception of *mediate* essence, by contrast, sets may have further features that arise from the chaining of immediate essence, and, in particular, sets can ‘inherit’ certain essential features from their members. For instance, assuming that Donald Trump is essentially the son of Fred C. Trump, it will turn out to be mediately essential to $\{$Barack Obama, Donald Trump$\}$ that it have a member that is the son of Fred C. Trump. And assuming that Donald Trump and Barack Obama are both essentially human, it is part of $\{$Barack Obama, Donald Trump$\}$’s mediate essence that it contain only human beings. But it is crucial to bear in mind that the inheritance of essential features is restricted to features that are (at least mediately) essential to the members of the relevant set. For instance, given that it is not essential to Donald Trump and Barack Obama that they be presidents in 2017, nor essential to Donald Trump that Barack Obama be president in 2017 or to Barack Obama that Donald Trump be president in 2017, it will not turn out to be even mediately essential to $\{$Barack Obama, Donald Trump$\}$ that it contain (some/only) presidents in 2017. Of course, this does not preclude that one can *pick out* the set $\{$Barack Obama, Donald Trump$\}$ in different terms than by its members, and, in particular, by the phrase ‘the set of all the US presidents in 2017’. But that I can pick out the set in this way merely means that the description is uniquely satisfied by the set, not that this is an essential feature of it.

With these considerations in the background, let us return to the case of set-nominalist DE and the property of charge. Recall that, according to this view, the property of charge would be identified with the set of all the (possible) objects that play role *R*. Call this set ‘*s*’. If we translate SDE’s claim that it is essential to charge that all of its instances play role *R* into set-nominalist terms, we arrive at the following claim: It is essential to *s* that all of its members play role *R*. But now, if we understand ‘essence’ in terms of *immediate* essence, then, given what was said before, this claim will turn out as straightforwardly false. For, in perfect analogy to the case of $\{$Barack Obama, Donald Trump$\}$ the only immediately essential features of *s* are that it be a set and that it contain these-and-that objects: electron *a*, electron *b*, a balloon that gets charged by being rubbed by child, a hair in dry air, and so forth. The natural follow up question is then: Might being such that all of its members play role *R* at least be a *mediately* essential feature of *s*?^17^

Let me first note that, even if it were, that would be a rather poor consolation for the proponent of set-nominalist DE. For if being such that all of one’s members play role *R* would be an essential feature that *s* has merely inherited from its members, the *ultimate* source of natural modality would not lie in the essences of properties, but rather, in the essences of particular objects. The proxy account would thus result in a radical shift of the view, and be a far cry from the original big picture of DE.

More importantly, however, we would plausibly not even get the intended result that *s* inherits the relevant essential feature—viz., that *s* is such that all of its members play role *R*—from its members. To see this, note first that it would be utterly implausible to hold that it should be essential to some of *s*’s members that another member of *s* play role *R*, e.g. essential to one specific electron that some other specific electron play role *R*. The only genuine option to consider for how the inheritance might work is thus that it is essential to *every* member of *s* that this member play role *R*. But while many philosophers with essentialist leanings would be happy to hold that electrons play role *R* essentially, for other members of *s*, the parallel essentialist claim looks simply implausible. Take, for instance, the balloon. That the balloon happens to be charged at some point in time due to external influences has nothing to do with what the balloon is at its very core. The balloon was not charged at some earlier point in time and it will cease to be charged in the future, we may assume, and it was possible for it to be never charged in the first place. So we can also rule out the option that *s* inherits the relevant essential feature from all of its members, and thereby the option that it is even mediately essential to *s* that all of its members play role *R*.

Nominalist DE thus seems to face a dilemma. If it admits proxy properties into the ontology, these properties will fail to have dispositional essences. And if it does not admit them, there are no candidates left to play the role of bearers of dispositional essence. It would thus seem that, on both horns, the Essentialist Claim cannot be salvaged, and thus no ‘full-blown’ account of nominalist DE can be provided. In fact, matters seem to get even worse: It would seem that any failure to account for the Essentialist Claim would threaten to further spill over to the Explanatory Claim. For, if there are no dispositional essences, how could there be any explanations of natural modality in terms of them? Thus, plausibly, if the Essentialist Claim has to go, so does the Explanatory Claim.

In what follows, however, I want to argue that there is a way out of the dilemma. The apparent difficulty of the first horn merely arises because we have a too narrow conception of essence in mind. By going beyond the nowadays common construal of essence exclusively in terms of *objectual* essence and instead invoking the notion of *generic* essence, the nominalist has an elegant and natural way to re-capture dispositional essence without any need for relying on proxy properties. The notion of generic essence has been introduced in the literature by Fabrice Correia (2006), and has recently become a subject of heightened interest in the literature (see e.g. Correia and Skiles (2019); Fine (2015); Rayo (2015)).^18^ Drawing on this literature, I will argue that there are strong independent reasons for countenancing the notion of generic essence (§4.2). I will then apply the notion to the dispositionalistic case, and develop my account of nominalist DE on its basis (§4.3).

### Generic essence to the rescue

Traditionally, one kind of question that essence has been seen as connected essense to are questions such as:^19^ ‘What is God, at her very core?’ ‘What is Socrates?’ ‘What is singleton {Socrates}?’ Thus, we have questions of the general form:

      (O) What is *a*? (with ‘*a*’ a singular term).

Answers to such questions would then be, for instance: ‘God is, by her very nature, almighty’, ‘Socrates is essentially human’ and ‘It is essential to singleton {Socrates} that it have Socrates as a member’. Essentialist talk of this sort is congenial to the objectual notion of essence that is common in contemporary metaphysics as described earlier. This objectual notion construes essence in terms of what is essentially true of some entity, the bearer of essence. For they can be perfectly brought into the canonical form:

      (O) It is essential to *a* that *p*.^20^

      $$\Box _{a}p$$.

However, questions and answers of this kind are not the only ones that have traditionally been associated with the notion of essence. Other questions that have been discussed are: ‘What is it, at its very core, to be human?’, ‘What is it to know a proposition?’, ‘What is it to be wise?’ Answers to these questions may then be expressed by sentences such as: ‘For someone to be human essentially involves for her to be rational’, ‘It is essential to knowing a proposition that one justifiedly believes it’, ‘To be wise is, at its very core, to know how to live well’. On the face of it, questions and answers of this kind are of the following form:

      (G) What is it to *F*? (with ‘*F*’ as predicate).

      To *F* essentially involves that *p*.^21^

Hence, we are confronted with a variety of essentialist claims whose surface form does not match the logical form of claims of objectual essence. Now, the obvious move at this point would be to try to cast such claims in the objectual form, by re-interpreting them as claims about the essence of ‘general’ entities such as properties or kinds. Following this idea, one may maintain that e.g. the first of the given examples is really of the following form: ‘It is essential to the property of being human that everyone who instantiates it is rational.’

A first, and rather obvious, disadvantage of this account, however, would be that, on the face of it, the sentence ‘for someone to be human essentially involves for her to be rational’ does not seem to ‘speak about’ the property of being human. The reconstruction of the essentialist claim in terms of properties would thus bring in an ontological commitment that seems to be absent in the original formulation. While this is certainly not a knock-down argument against this interpretation, it may still give us some first reason to be wary, and suggests that, other things being equal, it would be preferable to have an alternative account at our disposal. The second reason that tells against this account is more forceful. Even if we do assume a rich ontology of properties, kinds etc., we will not be able to interpret all (G)-cases in terms of them. Correia (2006) provides the example of the predicate ‘is a non-self-exemplifying property’. Arguably, ‘a non-self-exemplifying property, as such, is essentially many things: non-self exemplifying, a property, an abstract object, a non-self-exemplifying property, etc.’ (p. 762). But we cannot assume that there is a corresponding property of being a non-self-exemplifying property, on pain of getting into Russell’s paradox. Hence, no bearer of essence is available, irrespective of whether we grant an ontology of properties, kinds etc.. And thus, we have to find another way to account for such (G)-type essentialist truths.^22^

The core idea behind generic essence is now to take (G)-type essentialist claims at face value, rather than seeking to analyze them in terms of objectual essence. Thus, the friend of generic essence countenances a form of essence that matches the (G)-type as a further form of essence in its own right, i.e. as a different form of essence that is not reducible to objectual essence.^23^ In the case of generic essence, we thus have no entity of any sort (be it a particular, a property, a fact, or something else) which is the bearer of the essence. Instead, the essence concerns, so to speak, what certain ways for things to be are essentially like: that to be in a certain way essentially involves that one be thus-and-so.^24^ In formal language, generic essentialist claims can then be expressed via the $\boxminus _{F} p$-operator, which, in contrast to the $\Box _{e} p$-operator, takes *predicates*—rather than singular terms—as its subscript.^25^ Casting our previous example in this way will then give us:

      To be human essentially involves that one is rational.

      $\boxminus _{is \; human} \forall x (x$ is human $\rightarrow x$ is rational).

I take the considerations in this section to provide us with strong independent reasons for countenancing the notion of generic essence. In what follows, I will thus assume that we have this notion in our metaphysical toolkit, and construct the proposed account of nominalist DE on its basis. As we will see, endorsing the notion of generic essence allows us to provide very natural nominalist substitutes for SDE’s claims, which are not affected by the difficulty sketched in §4.1.

### The account of austere nominalist dispositional essentialism

Here are now the four components that jointly form the account of Austere Nominalist Dispositional Essentialism (‘ADE’), my proposal for an account of nominalist DE:

*Austere Ontology*

Following the insight that properties construed as sets of (possible) particulars would fail to have dispositional essences, the first element of the proposed account of ADE is an austere ontology with regard to properties. That is, the account does not appeal to any form of proxy properties such as sets of particulars, and rather maintains that there are no properties whatsoever.

*Fundamental Predicative Truths*

Moreover, the account does not incorporate only an austere ontology, but also an austere account of what I shall call ‘predicative truths’, that is, truths such as that that electron *a* is charged or that the ocean is blue.^26^ ADE takes certain predicative truths—such as, arguably, the truth that electron *a* is charged—as fundamental, rather than seeking to provide explanations in terms of something else, such as property instantiations, set memberships or resemblances between particulars.^27^ Expressed in formal language, we may thus have:

      $$\mathbb {F} (Fa)$$.

It goes without saying, however, that this does not mean that the account maintains that *all* predicative truths are fundamental. A proponent of the account may plausibly want to reject the idea that truths such as that the ocean is blue or that New York is a city are fundamental, just as property realists and proponents of other forms of nominalism would.

*Generic Dispositional Essence*

As already hinted at in the previous subsection, instead of construing dispositional essences as objectual essences of (sui generis or proxy) properties, the proposed account invokes the notion of generic essence to account for dispositional essence. Thus, returning to our example case, instead of saying that *it is essential to the property of charge* that charged objects play role *R*, the account simply says that *to be charged essentially involves* that one plays role *R*:^28^

      $$\boxminus _{F} (\forall x (Fx \rightarrow Rx))$$.

Hence, according to the account, there are no entities that are the bearers of dispositional essence. Instead, dispositional essence concerns what certain ways for particular objects to be essentially involve: that being thus-and-so essentially makes a particular object play a certain causal-nomological role.

In addition to a substitute for SDE’s specific essentialist claims, we also need a substitute for its general claim that *at least some of the fundamental properties* possess a dispositional essence. In the case of property realism, we have the first-order predicates ‘$\mathscr {D}$’ and ‘$\mathscr {F}$’, which apply to names for properties with a dispositional essence or fundamental properties, respectively. In the nominalist case, by contrast, we will need *second-order* predicates, i.e., predicates that apply to predicates. Let us use the symbols ‘$\mathscr {D}_{2}$’ and ‘$\mathscr {F}_{2}$’ for this. Now, in parallel to our understanding of $\mathscr {D}$ in the first-order case, we may take $\mathscr {D}_{2}$ to apply to some predicate *F* if being *F* essentially confers a certain causal-nomological role on all things that are *F*. With regard to fundamentality, recall that I suggested two ways in which one may want to understand the claim that a given property is fundamental. First, one may adopt a primitive conception of this notion. Or, second, one may take a property to be fundamental iff at least one instantiation of it is fundamental, in the sense of ungrounded. As our nominalist analogue of the former account of fundamentality, we can simply countenance the ‘$\mathscr {F}_{2}$’-notion as a primitive.^29^ And in analogy to the latter account, we may say that to *F* is fundamental iff there is at least one ungrounded truth of something’s being *F*. That is, in this latter case, we would have:

$$\mathscr {F}_{2} (F)$$ iff $$\exists x (\mathbb {F} (Fx))$$.

We may then express the nominalist equivalent of the Essentialist Claim as follows:

$$ \exists X (\mathscr {F}_{2} (X) \; \& \; \mathscr {D}_2 (X))$$.

Here, it is of crucial importance, however, how we interpret the second-order quantifier in this claim. For according to one common understanding of second-order quantification, the objectual interpretation, second-order quantifiers range over certain kinds of entities, such as properties, concepts or sets of particulars. And under this interpretation, the Essentialist Claim would bring us back to either property realism or a form of proxy nominalism.

The objectual interpretation of second-order quantification is not the only one available, however, and there are independent arguments that tell against it. Here is one such argument, due to Prior (1971): Plausibly, the ontological commitment of bound variables should line up with the commitment of the expressions replaced by the variables—uses of quantifiers should commit one at most to entities of the kind denoted by the expressions that the variables stand in for. But second-order variables stand in for predicates, and according to a widely held view, predicates do not have the semantic function of denoting entities of any kind (be they properties, concepts, sets of objects or whatever have you).^30^ Hence, the ontologically committal objectual interpretation should be rejected.^31^

The alternative option that I wish to suggest on behalf of ADE is to endorse a primitivist account of second-order quantification which does not seek to analyze second-order quantifiers in different terms, but rather countenances them as bits of primitive ideology (see Prior (1971); Rayo and Yablo (2001); Williamson (2003), and Wright (2007)).^32^ On this understanding, both first-order and second-order quantifiers are in essence means of generalization: devices that allow us to express more general facts about the world than we could otherwise communicate. Moreover, both kinds of quantification obey similar introduction- and elimination rules.^33^ In particular, in both the first-order and the second-order case, existentially quantified sentences are implied by their instances.^34^ And—pace the substitutional interpretation—in both cases, quantified sentences can be true even if the language lacks the means to express the instances. These similarities between first-order and second-order quantification notwithstanding, however, it should not come as a surprise that only first-order, but not second-order quantifiers incur ontological commitments, given that the bound variables occupy different syntactic positions and perform different semantic functions in both cases.

*Explanations of Natural Modality in Terms of Generic Dispositional Essence*

Turning to the substitute for SDE’s second core claim, the Explanatory Claim, matters prove to be even more straightforward as soon as we invoke generic essence. Starting from the account of SDE, all that we have to do is to replace objectual essence by generic essence, and leave all the rest as it stands.

      To see this, recall our toy-example in the case of SDE:

It is essential to charge that every charged object plays role *R* < every charged object plays role *R*.

$$\Box _{Fness} (\forall x (Fx \rightarrow Rx)) < \forall x (Fx \rightarrow Rx)$$.

It is essential to charge that every charged object plays role *R*, *a* is charged < *a* plays role *R*.

$$ \Box _{Fness} (\forall x (Fx \rightarrow Rx)), Fa < Ra$$.

By simply replacing objectual essence by generic essence, we obtain:

To be charged essentially involves that one plays role *R* < every charged object plays role *R*.

$$\boxminus _{F} (\forall x (Fx \rightarrow Rx)) < \forall x (Fx \rightarrow Rx)$$.

To be charged essentially involves that one plays role *R*, *a* is charged < *a* plays role *R*.

$$ \boxminus _{F} (\forall x (Fx \rightarrow Rx)), Fa < Ra$$.

And also in the case of the laws of nature, the space of options for ADE exactly matches the space of options for SDE. Just as proponents of SDE, proponents of ADE can choose to (a) identify the laws with certain dispositional essentialist truths, (b) identify the laws with generalizations that reflect the dispositional essentialist truths, (c) identify the laws with generalizations that are explanatorily powerful and simple, or, finally, (d) dispense with laws all together.

This concludes the exposition of the account of ADE. Note that, while my exposition invoked a rather specific regimentation of the Essentialist Claim and the Explanatory Claim, this is merely for means of presentation: in order to illustrate the ‘translation’ of SDE into the nominalist account in a precise way, and to be able to demonstrate that it indeed can be made to work. But in the end, nothing hinges on whether this exact form of SDE or another variant is taken as the point of departure. Thus, the proposed account of ADE affords an ultimately fully general ‘translation schema’ from any reified account of DE to a corresponding nominalist version.

With the view of ADE set out, in the remainder of the paper, I shall now explore the account in some more detail. In the following subsection §4.4, I will further clarify the relationship between ADE and three views on natural modality that bear important similarities to ADE, viz., SDE, primitivism about the laws of nature, and Causal Nominalism. And, finally, in subsection §4.5, I will show that ADE is immune to crucial problems that affect Causal Nominalism.

### Comparison to other views on natural modality

*Comparison to SDE*

Both SDE and ADE invoke essentialist truths as the ultimate source of natural modality. The difference between the two views consists in the fact that proponents of SDE postulate properties that are the bearers of the relevant essences, whereas ADE invokes generic essences that do not have a bearer. When asked ‘To which entity is it essential that negatively charged particles repel each other?’, the proponent of SDE will answer that it is essential to the property of charge, whereas the proponent of ADE will answer that this is not essential to any entity at all. austere nominalists can say that the given prejacent (i.e., the embedded content) is essential to what it is to be charged, but they will hasten to add that ‘what it is to be charged’ is not an expression denoting an entity. The essentialist truths of ADE and SDE do not differ with regard to their prejacents, by contrast. Proponents of both SDE and ADE can endorse the same logical form of the prejacents of the essentialist claims, and have the exact same range of options available regarding the kinds of natural modalities that they take to pertain to the essences (counterfactual, causal, primitive dispositional etc.). And the explanations of natural modality offered by ADE exactly mirror those given by SDE, except for the fact that generic essence replaces objectual essence. Moreover, given that generic essence implies metaphysical modality in the same way as objectual essence does, SDE and ADE will have perfectly analogous modal implications.^35^ In particular, both views will have it that an object’s being charged necessitates that it plays role *R*, and, depending on the embedded natural modality, yield the result that the fundamental predicative truths are not freely modally recombinable.^36^ SDE and ADE thus ultimately differ merely with regard to two features: first, concerning their commitment to properties, and second, with regard to the kind of essence they invoke—objectual essence in the case of SDE, and generic essence in the case of ADE.

The former difference is, I take it, one that should not come as a surprise. A nominalist account, as such, cannot incorporate any sui generis properties, but at best proxy properties. And whether an account incorporates proxy properties, that are, one might think, nothing but ‘shadows’ of the deeper underlying metaphysical reality, should plausibly not be seen as the hallmark of whether an account can be still considered as genuinely dispositionalistic or not. More importantly, as we have seen in §4.1, proxy properties would fail to have dispositional essences. And consequently, far from being something that turns an account into a form of DE, the incorporation of proxy properties would jeopardize the dispositionalist character of the account. Thus, if one is convinced that the commitment to properties is a necessary ingredient of DE, one is bound to regard nominalist DE as a lost cause right from the start, and there is nothing that I can do to convince them otherwise.^37^ My aim here can thus be no more than to offer an account for the philosopher who *is* willing to grant me that we can, so to speak, separate the dispositionalistic aspect of SDE from its ontological one, and explore matters further from there.

But how about the second difference, the one between objectual vs. generic essence? I am happy to grant that this change is a fairly substantial one. But it is my view that this change does not compromise the dispositionalistic aspect of ADE. Just as the proponent of SDE claims that ‘laws are not thrust upon properties, irrespective, as it were, of what those properties are’ (Bird 2007, p. 2), the proponent of ADE will say that the laws are not thrust upon ways for things to be, irrespective of what those ways are. While, according to SDE, nomic constraints arise from what the property of charge is at its very core, the proponent of ADE will say that they arise from what to be charged is at its very core. And this is, I take it, simply the very same idea ‘translated’ into the nominalist framework, and exactly what we should expect when we move from a property realist to a nominalist framework. While the property realist ultimately conceives of the world as populated by particulars and properties instantiated by particulars, the nominalist sees the world as one of particulars, characterized by *how they are*. For the property realist, the relevant essence concerns essential features of properties instantiated by particulars. For the nominalist, by contrast, the relevant essence should address essential features of how particulars are: that for a particular to be thus-and-so essentially involves being so-and-so. Construing dispositional essence in terms of generic essence is thus a move that is very natural and congenial to the broader underlying nominalist big picture.

All in all, the two differences between SDE and ADE should not be considered as ones that call the dispositionalistic character of the account into doubt. The account can provide substitutes for SDE’s two core tenets—the Essentialist Claim and the Explanatory Claim—that can naturally be incorporated into a nominalist framework, and that are still genuinely dispositionalistic in spirit. ADE is thus, one may say, really just SDE minus properties.

*Comparison to primitivism about the laws of nature*

Another question concerns ADE’s relationship to *primitivism about the laws of nature* (Carroll 1994; Maudlin 2007). Roughly, according to this view, lawhood is metaphysically ‘bedrock’ in the sense that no account of lawhood in different terms can be given. The common regimentation of the view employs a sentential law-operator, ‘*L*’, which is outfitted with an axiom to the effect that $L\phi$ necessitates $\phi$.^38^ The primitivist’s ideology thus strikingly mirrors ADE’s ideology. Both employ sentential operators (the *L* and the $\boxminus$-operator, respectively), such that $operator \; \phi$ is taken to necessitate $\phi$, and to thereby impose nomic constraints on the ‘mosaic’ of non-modal truths. What is more, ADE and law-primitivism share the same ontological commitments, or, better, lack thereof: in contrast to SDE, neither ADE nor law-primitivism invokes properties, or any other kinds of ‘lawmaking’-entities, in their accounts. And thus, one might wonder whether ADE amounts to a particular version of law-primitivism.^39^

To evaluate this question, we would need a precise criterion for what law-primitivism consists in, a matter which is not entirely clear in the literature. One possible criterion would be *ontological*: a theory of the laws of nature should be counted as a type of law-primitivism iff it is not committed to lawmaking entities.^40^ This criterion would nicely line up with the common classification of a variety of anti-Humean theories of the laws of nature. It would classify law-primitivism as law-primitivism (since it is not committed to any lawmaking entities), while classifying SDE, the nomic necessitation view (Armstrong 1983; Dretske 1977; Tooley 1977) and divine voluntarism (Foster 2004; Hildebrand and Metcalf xxxx) as not being types of law-primitivism (since they are committed to properties/nomic necessitation relations/god). However, the criterion would erroneously classify the Humean best system theory of laws (Lewis 1983; Beebee 2000; Loewer 2012), as well as Marc Lange’s (2009) account as law-primitivism. According to the Humean, the laws are those generalizations that are simple, explanatory powerful etc., and there is nothing deeper that governs or enforces these regularities. According to Lange, there are fundamental counterfactual truths, and the laws are roughly those regularities that are stable under counterfactual variation. Since neither one of the views comes with a commitment to specific entities, both of them would meet the ontological criterion for law-primitivism.^41^ However, Humeanism is commonly considered to be a paradigm of non-primitivism about laws. And while Lange’s view is indeed oftentimes labeled ‘primitivism’, it is labeled ‘primitivism *about counterfactuals*’, not ‘primitivism about *laws*’.

What seems to go wrong in the classification of the two accounts based on the ontological criterion is that, intuitively, neither Humeanism nor Lange take lawhood to be brute. Instead, they offer a reductive analysis of lawhood, i.e., an analysis of lawhood in terms of phenomena that bear themselves no essential or conceptual ties to lawhood: in terms of a generalization’s being explanatory powerful and simple, or stable under counterfactual variation, respectively. That these analyses do not involve any further entities is of no importance; they are reductive analyses all the same.^42^ Law-primitivism, by contrast, has it that no reductive analysis of lawhood can be given. These considerations suggest that we should not construe the relevant bruteness of lawhood in ontological terms, but, rather, simply in terms of a lack of reductive analysis: i.e., an account should be counted as a form of law-primitivism iff it takes lawhood not to be reductively analyzable.

In the case of ADE, as we have seen, there are basically two options for accounts of laws, apart from dispensing with laws all together: identifying the laws with (1) certain generalizations, or with (2) certain dispositional essentialist truths. No matter which way we go, however, lawhood will have a reductive analysis. On the former, generalizations-based approach, lawhood is analyzable in terms of whatever distinguishes the relevant regularities—such as their explanatory power and simplicity, or their reflecting a generic dispositional essence in the right way. And on the latter approach, lawhood is roughly analyzed in terms of the relevant truth’s being a generic dispositional essentialist truth of the right kind. To show that all of these analyses are reductive, it is sufficient to note that it is not part of the essence/concept of generic essence that generic essence be connected to lawhood. Recall that when generic essence was introduced in §4.2, lawhood was never mentioned. Furthermore, one can have an entirely adequate understanding of generic essence and yet deny that any generic essentialist truths express laws of nature. And clearly, not all generic essentialist truths correspond to laws of nature, as examples such as ‘$\boxminus _{is \; human} \forall x (x$ is human $\rightarrow x$ is rational)’ from §4.2 witness. It thus seems extremely plausible that any conceptual/essential connections between lawhood and generic essence pertain to the essence/concept of lawhood, rather than the essence/concept of generic essence. And thus, lawhood is not a brute status that resists analysis in different terms, and ADE does not turn out to be a form of primitivism about the laws of nature. Of course, none of this is to deny that ADE is a type of primitivism. It is. But it is primitivism about *generic essence*, not about laws. Indeed, SDE is also a form of primitivism in this sense: primitivism about *objectual essence*. So if one is not willing to sort SDE together with law-primitivism and Lange’s view merely on the basis of their invoking primitive ideology in their accounts, one should feel no temptation to do so with ADE either.

Still, one might wonder in how far the big pictures of ADE and law-primitvism really differ, apart from their treatment of lawhood. So let me conclude the discussion of law-primitivism by adding a bit more on this question. In the case of law-primitivism, the ‘governing’ *L*-truths that impose a nomic order on the world are in a sense ‘sui generis’: *all* *L*-truths are laws, and the *L* operator’s *only* metaphysical task is to account for lawhood. In the case of ADE—just as in the case of SDE—by contrast, the truths that create nomic connections are part of a broader phenomenon, viz. that of generic essence. As we have seen, not all generic essentialist truths are laws, and generic essence may serve many theoretical tasks unconnected to lawhood, such as figuring in an analysis of metaphysical modality, underlying grounding, and providing second-order identity conditions. Another aspect in which they differ is that, in the case of law-primitivism, the laws are in an intuitive sense a ‘global’ phenomenon: there is no particular aspect of the world that lawhood has its source in and thus lawhood belongs, so to speak, to the world as a whole. In the case of ADE—once again just as in the case of SDE—by contrast, lawhood may be seen as a ‘local’ phenomenon: the laws pertain to certain ways for things to be. And this difference is also witnessed in a crucial formal difference in the views’ ideologies. While the primitivst’s *L*-operator simply requires sentences as input, ADE’s and SDE’s essentialist operators have an additional slot for predicates or properties, respectively. Moreover, it also has the important consequence that ADE’s ‘space of options’ for the world’s lawhood-structure is larger than that of law-primitivism. Whereas SDE and ADE would distinguish between a ‘scenario’ in which Coulomb’s law pertains to the essence of (the property of being/what it is to be) charged vs. one in which it pertains to, say, the essence of (the property of being/what it is to be at a certain) distance, for law-primitivism these two scenarios would collapse into a single scenario in which Coulomb’s law is a primitive law of nature. Metaphorically speaking, in order to figure out the complete nomic structure of the world, more work is needed in the case of ADE and SDE than in the case of law-primitivism. While the primitivist could call it a night after figuring out the embedded content, the friends of ADE and SDE would have to go on to answer the ‘tagging’-question.^43^

*Comparison to Causal Nominalism*

Finally, let us turn to the relationship between ADE and Whittle’s Causal Nominalism (2009), the only other extant proposal for a nominalist view in the vicinity of Dispositional Essentialism. If construed in terms of ground, Causal Nominalism can be regarded as a combination of three characteristic claims. First, as already mentioned in §4.1, Whittle adopts the strategy of identifying properties with the sets of (possible) objects that *play a certain causal/counterfactual role*. So for instance, we plausibly have on her account:

charge = the set of all (possible) objects which play role *R*.^44^

Second, Causal Nominalism maintains that predicative truths obtain in virtue of the corresponding truths regarding counterfactual roles. That is, according to Causal Nominalism, we would have:

$$Ra < a$$ is charged.

And third, the account takes certain counterfactual truths to be fundamental. Thus, if *a* is some electron, we may have:

$$\mathbb {F} (Ra)$$.

Causal Nominalism arguably shares SDE’s idea that properties are intimately tied to causal-nomological roles. But it parts way with both SDE and ADE in two critical respects. First, as we have seen in section §4.1, properties construed as sets fail to have dispositional essences. Hence, pace Whittle, it is not the case that, on the account, ‘the functional role of a property is essential to it’ (p. 259). And the view does not incorporate ADE’s nominalist version of the Essentialist Claim either. Causal Nominalism thus drops the idea of dispositional essence from the picture. Second, Causal Nominalism reverses the order of explanation compared to SDE and ADE. While the latter two views maintain that *a* plays role *R* (in part) *because* *a* is negatively charged, Causal Nominalism maintains the opposite claim that *a* is negatively charged *because* *a* plays role *R* (cf. p. 266). And this reversal in the order of explanation clearly constitutes a major change in the account. Moreover, by taking counterfactual modality as brute, Causal Nominalism forfeits DE’s explanatory project of accounting for them in terms of dispositional essence.

All in all, although there are indeed some commonalities between Causal Nominalism on the one hand, and SDE and ADE on the other, the differences seem to prevail. While ADE might be fairly regarded as simply ‘SDE minus properties’, Causal Nominalism seems to provide us with an independent view of its own, which combines primitivism about counterfactuals (akin to Lange’s account) with dispositionalistic tendencies.

### Why ADE is immune to the problems that causal nominalism faces

As I have already indicated in the introduction, Causal Nominalism faces objections due to Tugby (2016). And since Causal Nominalism and ADE are both nominalist accounts in the vicinity of SDE, one might wonder whether ADE is subject to the same problems. In this section, I shall conclude my discussion of ADE by arguing that it is not, and that the problems raised by Tugby should thus give us no reasons to be sceptical about the prospects of ADE. In my discussion of these problems, I will construe matters in a slightly different way than Tugby does, however, and I will also add further considerations of my own.

The problems for Causal Nominalism can best be conceived of as a dilemma. The two horns of the dilemma arise corresponding to two ways of how the fundamental counterfactual truths can be construed in more detail on Whittle’s account: as *general* or as *particular*. On the general approach, we would directly take the truth ‘*Ra*’ to be fundamental, that is, a truth of the form:

For all objects *y* and magnitudes *u*, *v*:

If *a* were at distance *u* from *y* and *y* had charge *v*, *a* would exert a force of $\epsilon \frac{e \cdot v}{u^{2}}$.

On the particular understanding, by contrast, we would have various particular instances of *Ra* in place of *Ra* on the fundamental level, that is, a multiplicity of truths of the form:

If *a* were at distance $d_{1}$ from $b_{1}$ and $b_{1}$ had charge $c_{1}$, *a* would exert a force of $\epsilon \frac{e \cdot c_{1}}{d_{1}^{2}}$,

where ‘$b_{1}$’ designates a specific electron, and ‘$c_{1}$’ and ‘$d_{1}$’ specific magnitudes of charge and distance, respectively. But no matter which of these two options are chosen, problems arise.

The problem for the general approach is rather straightforward. On such a construal, we would be confronted with a universally quantified fundamental truth, in violation of the principle that all universal generalizations are grounded in their instances plus maybe totality truths. This principle is consensus in the debate on grounding, and it is part of all formal theories of ground. Giving up on this principle would thus come at a high cost: It would mean that we would have to revise core parts of our understanding of grounding, while it is yet unclear what might replace said principle.^45^

Turning to the particular accounts, the problem that emerges is this: If we take particular, rather than general natural modalities to be fundamental, we are confronted with a striking regularity with regard to the fundamental counterfactual truths: When we have a case in which some of the instances of *Ra* ‘come together’, we typically have a case in which *all of them* do. Thus, for instance, if some object *a* is such that, if it were in distance $d_{1}$ to $b_{1}$ and $b_{1}$ had charge $c_{1}$, *a* would exert a force of $\epsilon \frac{e \cdot c_{1}}{d_{1}^{2}}$, *a* will be typically also such that, if it were in distance $d_{1}$ to $b_{2}$ and $b_{2}$ had charge $c_{1}$, *a* would exert a force of $\epsilon \frac{e \cdot c_{1}}{d_{1}^{2}}$, etc.

On the face of it, if there were no deeper metaphysical explanation for the ‘clustering’ of all these counterfactuals, this regularity would look like something just extremely unlikely, like a form of ‘cosmic coincidence’. But now, it is unclear what would possibly explain their coming together on this particularist version of Causal Nominalism. Given that, on the proposal we are considering now, all these counterfactual truths are separate fundamental truths, we cannot account for them in terms of something further that grounds all of them. Nor can we explain the regularity in terms of the dispositionalist characterization of charge. For this characterization does not specify that, if we have one (or some) of the counterfactuals, we also have the others.

It thus seems that the only way to go would be to just bite the bullet and claim that this is just the way it is: There is simply no deeper explanation for the truth that, in our world, counterfactuals tend to come this neatly together. We just happen to be lucky and live in a particularly regular world in this respect. And, indeed, this move is exactly what a proponent of the Humean account of natural modality would say. Borrowing this move from the Humean, however, would come at a high dialectical coast for Causal Nominalism. It would deprive the resulting account from the core advantage that DE as a view among the anti-Humean variety of accounts of natural modality is commonly taken to have: that, unlike the Humeans, they *can* provide ‘genuine’ explanations for the regularities regarding fundamental truths, rather than conceiving of this uniformity just as a happy coincidence.^46^

Moreover, there is a second problem for Causal Nominalism that arises at least on the latter ‘particularist’ horn, but potentially also on the former ‘generalist’-horn, and it is this: Predications that are commonly considered as paradigm cases of intrinsic predications—such as our standard example of *a*’s being charged—will turn out to be extrinsic on Whittle’s account. For on the account, whether *a* is charged is not merely due to what *a* is like, but, rather, how other, wholly distinct, objects are: How $b_{1}$ would interact with *a*, how $b_{2}$ would interact with *a*, etc. If we want to go beyond intuitions and lend further support to the extrinsicality verdict, we may employ one of the accounts of extrinsicality that have been proposed in the literature, such as Gideon Rosen’s (2010) grounding-based account. On his account, we have:

*a* is *F* in an extrinsic fashion iff$_{\tiny {\text{ def }}}$ the fact that *a* is *F* is grounded in a fact that has an object *y* as a constituent which is not a mereological part of *a*.^47^

The account relies on the existence of facts, to which I do not wish to commit here, and it construes grounding in terms of a relation between facts, rather than in terms of a sentential connective. But to keep matters simple, let us play along, by assuming for heuristic purposes that we have facts in our ontology and extending our operationalist notion of grounding to a notion of fact-grounding in the obvious way.^48^,^49^ Then, Rosen’s account will yield the result that *a* is charged in an extrinsic fashion, as long as there is at least one object *b* that is not a part of *a* and interacts with *a* in the relevant way (such as, in our case, another electron which is not physically isolated from *a*). For in this case, the specific counterfactual ‘If *a* were at distance $d_{1}$ from object *b* of charge $c_{1}$, *a* would exert a force of $\epsilon \frac{e \cdot c_{1}}{d_{1}^{2}}$’ will be a partial ground for the general counterfactual *Ra*, which, on Whittle’s account, is in turn a ground for *a*’s being *F*. Hence, the specific counterfactual will also be a (mediate, partial) ground for *a*’s being *F*. And thus, *a* will turn out to be *F* in an extrinsic fashion.^50^

To summarize the results from the discussion: By flipping the explanatory direction and taking counterfactual truths to be fundamental rather than to be explained by essential truths, Causal Nominalism faces severe problems. If the view takes the relevant general counterfactuals to be fundamental, it conflicts with the commonly held belief that all universal generalizations are grounded in their instances. And if it takes the particular ones to be fundamental, it fails to account for certain regularities concerning natural modalities, and predications that are commonly taken to be paradigm cases of intrinsic predications turn out as extrinsic. ADE, by contrast, evades all of these problems.

First, the proponent of ADE has a very natural explanation for the regularity with regard to the ‘coming together’ of the particular counterfactuals at her disposal. Recall that the account takes the general counterfactual truth *Ra* to be grounded in the two truths $\boxminus _{F} (\forall x (Fx \rightarrow Rx))$ and *Fa* taken together. But now, it should be clear that the two truths $\boxminus _{F} (\forall x (Fx \rightarrow Rx))$ and *Fa* together not only provide an explanation for *Ra*, but also for all its instances, i.e., the particular counterfactuals. And consequently, the account can provide us with an explanation for the ‘clustering’ of these particular counterfactuals which, so to speak, is an explanation along the lines of a ‘common cause’-explanation: all of the counterfactuals are grounded in the very same two truths.

Second, the view does not give rise to a violation of the principle that all universal generalizations are grounded in their instances. It is indeed true that, on the account, the general counterfactual truth *Ra*—which has the form of a universal generalization—is immediately grounded in something else than in its instances—viz., in the two truths $\boxminus _{F} (\forall x (Fx \rightarrow Rx))$ and *Fa* taken together. But all that we have to say in order to preserve harmony with grounding orthodoxy is that *Ra* is not merely fully grounded in the two truths $\boxminus _{F} (\forall x (Fx \rightarrow Rx))$ and *Fa* taken together, but *also* fully grounded in its particular instances (plus maybe a totality truth). For the standard view is not that the *only* full ground for a universal generalization is given by its instances (plus maybe totality truths), but only that *one* full ground is.^51^

And third, it is straightforward to see that ADE does not yield the result that *a* is charged in an extrinsic fashion. On the proposed account, *a*’s being charged is taken to be a fundamental truth, that is, not grounded in anything. And *eo ipso*, it is not grounded in a truth that ‘involves’ an object that is not part of *a*. *a* thus turns out to be charged in an intrinsic fashion, just as it should be.

All in all, there is thus no need to worry that, as an account of nominalist DE, ADE is automatically also subject to the problems for Causal Nominalism raised by Tugby. These problems are not consequences of combining nominalism with dispositionalistic elements as such, but, rather, merely consequences of the specific way in which Whittle sets up her account. Hence, they should not deter us from adopting ADE.

## Conclusion

What I hope to have shown in this paper is that the combination of Dispositional Essentialism with nominalism is a perfectly coherent and tenable position that deserves further exploration. Abstracting away from the details of my discussion, we can recognize a simple and straightforward ‘construction-plan’ for devising a nominalist account out of a reified account of Dispositional Essentialism. Roughly, all that we have to do is to replace the objectual dispositional essences of properties with the corresponding generic dispositional essences, and to preserve the common explanatory hierarchy. The resulting account does not give rise to any of the problems faced by Whittle’s Causal Nominalism, preserves the core tenets of Dispositional Essentialism, and carries no commitment to anything but particulars. Thus, contrary to first appearance, dispositional essentialism can be combined with nominalism. The Dispositional Essentialist is free to choose whether she wants to countenance properties in her ontology, or to make do without them.

## Footnotes

1 Accounts of DE, broadly construed, include: Bird (2007); Harré (1970); Harré and Madden (1975); Ellis (2001); Ellis and Lierse (1994); Martin (2007); Molnar (2003); Shoemaker (1980) and Williams (2019).

2 See also Yates (2013) for arguments in favor of the claim that proponents of DE should employ a Finean rather than modal account of essence.

3 For overviews of the debate on grounding, see Bliss and Trogdon (2014); Correia and Schnieder (2012), and all the articles in Raven (2020).

4 See, however, Schaffer (2012) against transitivity, Thompson (2016) against asymmetry, and Jenkins (2011) against irreflexivity.

5 See e.g. Correia (2010); Dasgupta (2017); Fine (2012) and Schnieder (2010) for the operationalist formalization. I use this formalization in order to stay neutral regarding the existence of facts and propositions.

6 For discussions of entity-fundamentality, see e.g. Bennett (2017); Morganti (2020), and Tahko (2018). See Wang (2019) for an argument against interpreting entity-fundamentality in the context of DE in terms of ontological independence.

7 I am assuming here that the realist about properties thinks that predicative truths (such as the truth that some specific electron *a* is charged) are grounded in the corresponding truths about property-instantiations (such as that *a* instantiates charge). This view is commonplace among property realists, but see Dixon (2018) for arguments that the grounding goes in the opposite direction. In this case, we would plausibly instead have: $\mathscr {F} (F$ness) iff$_{\tiny {\text{ def }}} \exists x \; \mathbb {F} (Fx)$.

8 Here is a tentative proposal of how we might roughly understand this in more precise terms: There is no reductive analysis of all (truths involving) properties to solely (truths involving only) entities of other categories, such as e.g. sets of particulars.

9 Note that the qualification ‘fundamental’ in the characterization plays a crucial role and could not be omitted. Many philosophers without dispositionalistic inclinations would nevertheless be happy to countenance the existence of non-fundamental properties with dispositional essences such as e.g. water-solubility and fragility.

10 Some readers might wonder why I use first-order variables to stand in for properties, rather than second-order variables. I take, however, second-order quantification to be ontologically non-committal, and first-order quantifiers to range over entities of all ontological categories. See §4.3 for more on this take on quantification.

11 While most proponents of SDE endorse essentialist claims along these lines, and merely disagree regarding the question of whether we should invoke a counterfactual, causal or dispositional modality in the essences, this idea might be challenged. Thus, for instance, conservation laws might call for essentialist truths whose embedded content is non-modal. Such an account of SDE is further developed by Yates (2013). I focus on this standard version of SDE for means of illustration, but it should be clear how the considerations presented in what follows could be adopted to other accounts. Thanks to a reviewer for raising this issue.

12 One may think that, letting ‘*I*’ stand for instantiates’, we instead have: $\Box _{Fness} \forall x (xI F$ness$\rightarrow Rx)$. Likewise, there are two ways of construing the content of *R*: a predicative way and a way in terms of property instantiations. Which of the two alternatives is endorsed will not make a substantive difference for anything in what follows, but see also the later footnote 28.

13 In particular, one alternative option would be to construe the explanation in terms of essentialist explanation rather than ground (see Glazier (2017)). For further alternatives and related discussion, see Emery (2019); Hildebrand (2020) and Wilsch (2021).

14 This claim would be an instance of the more general principle of *Essence Grounds Prejacents*, according to which, for any *e* and *p*, $(\Box _{e} p) < p$. See Dasgupta (2014), Glazier (2017); Kment (2014); Rosen (2010); Vogt (yyyy), and Zylstra (2019) for discussion.

15 An alternative would be to think that the essentialist truth serves not as a first-order ground, but rather as a meta-ground, i.e., as something that grounds the grounding (see Bennett (2017), chapter 7; Dasgupta (2014, 2019) for discussion). On such an account, we would have: $Fa < Ra$ and $\Box _{Fness} (\forall x (Fx \rightarrow Rx))< (Fa < Ra)$. Anything that will be said in what follows could be adapted to the meta-grounding proposal in a straightforward way.

16 Alternatively, we might identify properties with functions that map worlds to the sets of objects that play the relevant roles in these worlds, i.e., with sets of pairs of worlds and sets of objects. I focus on the simpler account for means of presentation, but nothing in what follows will hinge on this choice. A further proposal that has been made is to identify properties with the mereological sums, rather than the sets of their instances. However, this proposal does not look promising (cf. Rodriguez-Pereyra (2008)), and the account would be subject to the same objections that I raise for the account of properties in terms of sets at a later point in this subsection.

17 I would like to thank a reviewer for suggesting the idea that proponents of set-nominalist DE might resort to the idea that sets inherit the relevant essential features from their members to me.

18 Note that the expression ‘generic’ here is meant in a different way than the notion of generics as discussed in philosophy of language and linguistics, which concerns sentences such as ‘tigers have stripes’, ‘mosquitoes carry malaria’ and ‘the dodo is extinct’.

19 My exposition of the reasons in favor of generic essence draws on the discussion in Correia (2006).

20 Other ways to formulate such claims include: ‘ It is true in virtue of the nature of *a* that *p*’, ‘It is part of the essence of *a* that *p*’, and ‘*a* is by its very nature such that *p*’.

21 Other ways to formulate such claims include: ‘It is true in virtue of what it is to *F* that *p*’ and ‘For something to be *F* essentially involves for it to be such that *p*’.

22 For further arguments that we cannot understand all cases of form (G) in terms of the essence of general entities, see Correia (2006) and Fine (2015). Moreover, see Correia (2006) for an argument that we cannot interpret the cases as merely reflecting the meaning of the relevant predicates.

23 Instead of countenancing two distinct primitive kinds of essence—objectual and generic—the proponent of generic essence can alternatively analyze objectual essence in terms of generic essence. On such an account, the aforementioned claim regarding the essence of Socrates would be e.g. rephrased as: It is essential to being (identical to) Socrates that one is human. See Correia (2006) and Fine (2015) for discussion.

24 The ‘ways’-idiom here should not be understood as committing one to ways as a sort of entities, and merely serves me as a means of imitating higher-order talk in natural language.

25 See Correia (2006) and Rayo (2015) for this formalization. For an alternative formalization in terms of a sentence-operator that binds free variables, see Fine (2015) and Correia and Skiles (2019).

26 Predicative truths as understood here are thus *not* meta-linguistic truths such as the truth that the predicate ‘is charged’ applies to ‘*a*’. They can be *expressed by using* predicates, but they are not *about* predicates.

27 The austere account of predicative truths has an arguably somewhat bad reputation in the literature. It has been labelled ‘Ostrich Nominalism’ and accused of skirting the task set for the nominalist rather than providing a solution (see Armstrong (1978)). However, the proponent of the austere view does not refuse to answer the question ‘What explains certain ‘basic’ predicative truths such as that *a* is charged?’, but, rather, she answers the question by saying ‘nothing’. Every account has to either assume that certain truths are fundamental, or assume an infinite descending grounding chain—the latter being a position that few would be willing to endorse. To make a point against the account, one would thus have to show that predicative truths are somewhat ill-suited to play the role of fundamental truths. I personally find convincing arguments to this effect wanting, but this paper is not the place where I can discuss these issues in due detail. If readers are already convinced that the austere account of predicative truths is untenable, I will not be able to convince them otherwise here. See e.g. Armstrong (1980); Peacock (2009) and Rodriguez-Pereyra (2002), chapter 3, for arguments against the austere account, and Devitt (1980); Melia (2005), and Cleve (1994) in favor of it.

28 Coming back to the discussion in footnote 12: If the proponent of SDE were to endorse versions of the essentialist claim that invoked ‘*xIF*-ness’ rather than ‘*Fx*’—i.e., have $\Box _{Fness} (\forall x (xIFness \rightarrow Rx))$ rather than $\Box _{Fness} (\forall x (Fx \rightarrow Rx))$—the shift from SDE to ADE would also include a modification of the former to the latter. Likewise, if *R* were assumed to be given in terms of property instantiations rather than in predicative terms, we would also have to correspondingly modify *R*. This would make the shift slightly more extensive, but still straightforward.

29 See e.g. Dorr & Hawthorne 2013 and Jones 2018 for this idea.

30 Austere nominalists have to maintain that predicates are non-denoting expressions, regardless of their stance on second-order quantification, on pain of having to go error theorist/fictionalist about all predicative sentences. But also many property realists will agree that predicates do not refer, based on considerations in the philosophy of language (such as, in particular, the notorious Concept Horse Paradox). See MacBride (2006) for a discussion of predicate reference.

31 See also Rayo and Yablo (2001) and Wright (2007) for a discussion of the argument as well as arguments in the vicinity. Here are two additional considerations that tell against the objectual interpretation. First, on the face of it, natural language seems to contain various quantificational expressions which do not seem to range over entities either, such as the ‘somehow’ in ‘I hurt him somehow, viz., by treading his toe’, and the ‘however’ in ‘However he says things are, thus they are’ (cf. Prior (1971)). And second, on the objectual interpretation, the expressive power of second-order quantification would need to be severely limited, on pain of paradox (cf. Rayo and Yablo (2001) and Williamson (2003)).

32 While all the mentioned proponents of the view agree that second-order quantification is primitive in the sense of ‘not analyzable in terms that do not invoke higher-order quantification’, there is significant disagreement regarding the extent of this primitivity: e.g. whether the semantics and meta-logics of second-order languages must be couched in higher-order terms too and in how far we can imitate second-order quantification in natural language. See Dunaway (2013) and Turner (2015) for an overview.

33 See Wright (2007) for further discussion of these rules.

34 That is, both $\exists x Fx$ and $\exists X Xa$ are implied by *Fa*.

35 See Correia (2006) for the idea that generic essentialist truths necessitate their prejacents in the same way as objectual essentialist truths do.

36 See Bird (2007) for an exploration of the modal consequences of SDE’s essentialist claims in the case of an embedded counterfactual.

37 Relatedly, I will not be able here to convince the philosopher who insists that, for an account to count as genuinely dispositionalist, powers (or maybe facts that have powers as constituents), have to act as truthmakers for statements regarding natural modality. But presupposing such an account of truthmaking would at least come close to being question-begging against the proponent of austere nominalism.

38 For this regimentation, see Hildebrand (2013) and Schaffer (2016).

39 Thanks to a reviewer for suggesting this idea to me.

40 Thanks to the same reviewer for comments suggesting this criterion.

41 One might think that Humeanism and Lange’s account are committed to lawmaking entities, viz., to the facts in the Humean mosaic and counterfactual facts, respectively. However, neither one of the views requires a reification of facts, since the views can be equally cashed out entirely on the level of truths (by means of a sentential grounding-connective, quantification into sentence-position etc.). The question of whether a proponent of these views countenances facts in her ontology is orthogonal to the question of which account of lawhood she endorses.

42 Broadening the focus from the debate on the laws to other areas of philosophy, we can see that parallel situations abound. To give just two examples, to my knowledge, it has never been suggested that accounts that analyze a given phenomenon, such as essence or intrinsicality, in modal terms should still be considered to be primitivist regarding the relevant phenomenon as long as they only refrain from reifying possible worlds. And the ‘justified true belief’-theory of knowledge is generally regarded as non-primitivist, even if a proponent of it stops short at claiming that to know a proposition is to justifiedly truly believe it, without postulating a relation of belief between the believer and the believed proposition, a property of being true etc.

43 In the parallel debate on the laws of metaphysics, Jonathan Schaffer (2017) takes this ‘tagging’-question, as well as the question of how finely grained distinctions between scenarios are drawn, to be exactly the crucial difference between ‘sui generis metaphysical laws’ vs. essentialist truths.

44 Whittle also suggests a version of Causal Nominalism (p. 246ff.), in which *R* would be replaced by a ramsified version of it. I focus on the non-ramsified version of Causal Nominalism for the sake of simplicity, but my discussion would equally apply to the ramsified version.

45 Tugby raises a different worry for this horn of the dilemma: He argues that general truths of natural modality would look suspiciously like laws of nature. However, this argument strikes me as problematic in two respects. First, while the relevant counterfactuals are general in certain respects, they are still particular in the sense of concerning what would happen *to this particular electron* *a*—in contrast to laws of nature that are fully general. And second, even if they did, it would be unclear why it should be worse for Whittle to take laws of nature rather than more specific counterfactual truths to be fundamental: in both cases, we would not get a reduction of all natural modalities on her account.

46 See also Filomeno (2019) for recents arguments to the effect that the ‘cosmic coincidence worry’ poses a serious threat for Humeanism.

47 I have modified Rosen’s definition in two respects in order to adopt it to the case at hand. First, Rosen’s account concerns property instantiations rather than predicative facts. And, second, Rosen’s account defines the global notion of an extrinsic property rather than the local notion of something having a property in an extrinsic fashion. Both modifications are straightforward and common, however. Rosen’s account is certainly not uncontentious, but so is any other account of extrinsicality that has been proposed thus far (cf. Marshall and Weatherson (2013)).

48 Thus, we would have: The fact *f* grounds the fact *g* iff$_{\tiny {\text{ def }}}$: $\exists p, q ((p<q$) & (*f* = the fact that *p*) & (*g* = the fact that *q*)).

49 The reliance on facts in Rosen’s account could arguably be circumvented by adopting some further modifications. Here is a tentative proposal: *a* is *F* in an extrinsic fashion iff$_{\tiny {\text{ def }}}$ $\exists p, \exists b \; (p <^{par} Fa \; \& \; \lnot (b \sqsubseteq a) \; \& \; b \triangleleft p$). Here, ‘$<^{par}$’ stands for partial grounding, ‘$\sqsubseteq$’ for (proper or improper) mereological parthood, and ‘$b\triangleleft p$’ for a notion that might be understood along the lines of ‘*b* occurs in *p*’ or ‘*p* is about *b*’. This latter notion might be in turn either taken as primitive, or as defined as: $\exists X ($For *p* to be the case just is for *Xb* to be the case).

50 Does the problem also arise on the generalist horn of the dilemma? This is not clear, I think. On Rosen’s account, it crucially depends on whether we maintain that a universally quantified claim has all objects whatsoever as its constituents or not, which is a contentious issue.

51 See e.g. Rosen (2010) for the idea that universal truths can be grounded in other truths than merely their instances, and, in particular, in essentialist truths. One might worry that the proposed account gives rise to metaphysical overdetermination. For, on the account, the general counterfactual truth *Ra* would be taken to be both immediately grounded in the truths $\boxminus _{F} (\forall x (Fx \rightarrow Rx))$ and *Fa* taken together, and mediately grounded in them ‘via’ the instances of *Ra*, plus maybe a totality truth. Be that as it may, similar cases of grounding-overdetermination are in fact widespread, and should thus not be regarded as worrisome. For a particularly simple case, take any truth of the form $(p \wedge q) \vee p$. Any such truth is both immediately grounded in *p*, and mediately grounded in *p* and *q* taken together.
